# Identification, expression, and artificial selection of silkworm epigenetic modification enzymes

**DOI:** 10.1186/s12864-020-07155-z

**Published:** 2020-10-23

**Authors:** Rui Gao, Chun-Lin Li, Xiao-Ling Tong, Min-Jin Han, Kun-Peng Lu, Shu-Bo Liang, Hai Hu, Yue Luan, Bi-Li Zhang, Yan-Yu Liu, Fang-Yin Dai

**Affiliations:** State Key Laboratory of Silkworm Genome Biology, Key Laboratory of Sericultural Biology and Genetic Breeding, Ministry of Agriculture and Rural Affairs, College of Biotechnology, Southwest University, Chongqing, 400715 China

**Keywords:** Domesticated traits, Epigenetic modifying enzymes, Selective sweeping, *BmSuv4–20*, *BmDNMT2*, Reproduction, Silkworm

## Abstract

**Background:**

Understanding the genetic basis of phenotype variations during domestication and breeding is of great interest. Epigenetics and epigenetic modification enzymes (EMEs) may play a role in phenotypic variations; however, no comprehensive study has been performed to date. Domesticated silkworm *(Bombyx mori)* may be utilized as a model in determining how EMEs influence domestication traits.

**Results:**

We identified 44 *EMEs* in the genome of silkworm (*Bombyx mori*) using homology searching. Phylogenetic analysis showed that genes in a subfamily among different animals were well clustered, and the expression pattern of *EMEs* is constant among *Bombyx mori*, *Drosophila melanogaster*, and *Mus musculus*. These are most highly expressed in brain, early embryo, and internal genitalia. By gene-related selective sweeping, we identified five *BmEMEs* under artificial selection during the domestication and breeding of silkworm. Among these selected genes, *BmSuv4–20 and BmDNMT2* harbor selective mutations in their upstream regions that alter transcription factor-binding sites. Furthermore, these two genes are expressed higher in the testis and ovary of domesticated silkworm compared to wild silkworms, and correlations between their expression pattern and meiosis of the sperm and ova were observed.

**Conclusions:**

The domestication of silkworm has induced artificial selection on epigenetic modification markers that may have led to phenotypic changes during domestication. We present a novel perspective to understand the genetic basis underlying animal domestication and breeding.

**Supplementary information:**

**Supplementary information** accompanies this paper at 10.1186/s12864-020-07155-z.

## Background

Domestication of plants and animals is a milestone in the history of human civilization that involves the long-term artificial selection of advantageous natural variations. The history and genetic basis of rapid phenotypic evolution in domestication and breeding remains a topic of research interest. Epigenetics is usually defined as stably heritable phenotypes resulting from several types of changes on chromosomes such as DNA methylation, histone post-translational modifications (PTMs), non-coding RNA regulation, and chromatin remodeling, but not on the DNA sequence [1]. Epigenetic modifications can alter the structure of chromatin and play a critical role in maintaining genomic integrity, gene expression, cellular memory, cell reprogramming [2, 3], and individual development [4, 5]. Epigenetic modification enzymes (EMEs) establish and erase marks on chromatin, along with specific “readers” and together comprise the dynamic “histone code,” which determines the diversity of epigenetic space and the complexity of gene expression regulation. Prior reports have shown that the genomic regions containing *EMEs* had undergone selection during the domestication of several animals (Additional file 1: Table S1). Meanwhile, epigenetic modifications were also determined to be involved in the regulation of domesticated phenotypes [6]. These studies suggest an impact of domestication on epigenetics, but the mechanism of epigenetics and the function of EMEs regulating domesticated traits requires additional investigations.

Insects have recently emerged as models for epigenetics studies and have been shown to harbor a different epigenetic model compared with vertebrates. Most insects display a sparse epigenomic map, and the methylated sites are found almost exclusively in gene bodies. The composition and function of the DNMT toolkit in insects also differ from those of mammals [7]. Kucharski et al. silenced the expression of DNA methyltransferase *Dnmt3* in honey bee larvae, which led to a dramatic shift from worker to queen developmental fate [8]. Subsequent studies have further suggested that epigenetic and environmental factors play a role on caste fate and social behavior plasticity in eusocial insects [9]. For example, CBP-mediated histone acetylation in the ant central brain can regulate caste-specific foraging and scouting behaviors [10]. In addition, extensive functional mutation studies confirmed that EMEs and are widely involved in the regulation of *Drosophila* lifespan [11], development [12], reproduction [13], sex-determination [11], learning, and memory [14]. Epigenetic modifications have also relatively been rare studied in other insects such as *Phenacoccus solenopsis* [15], *Nilaparvata lugens* [16], and *Tribolium castaneum* [17], providing novel insights on the model insect, *Drosophila*. Thus, additional studies on epigenetics in traditional non-model insects are necessary to reveal the developmental and evolutionary significance of epigenetic inheritance in insects.

Domesticated silkworm *(Bombyx mori)* is famous for spinning silk cocoons and thus is considered as an economically significant insect. It also serves as a model for lepidopterans and has been shown to be well suited for genetic research. *B. mori* was fully domesticated from *B. mandaina*, with a history of 5000 years of domestication and more than 150 years of breeding. Long-term artificial selection has definitely changed the domesticated silkworm in terms of economic traits related to silk production and a series of physiological and behavioral traits such as body color, docility, moving ability, disease resistance, and reproductive characteristics compared with *B. mandaina*. Reports have shown that the epigenetic pattern of silkworm has changed during domestication. Xiang et al. investigated the silk gland DNA methylomes of domesticated and wild silkworm and showed that DNA methylation levels are significantly elevated in *B. mori* compared with *B. mandaina* [18, 19]. In addition, several epigenetics modifications are involved in the regulation of silkworm development [20], reproduction, and sex determination [21, 22]. Thus, we hereby explored the potential use of silkworm as a model for studying the influence of epigenetics on phenotypic shaping during the domestication process. We initially raised the following questions: Are EMEs selected during silkworm domestication and breeding? If so, which type of epigenetic marker was under selection and whether it play a role in the change of domesticated traits?

The present study aimed to identify *EMEs* in the whole genome of silkworm, analyze their phylogenetic relationship in animals, and assess their tissue expression patterns. With the artificial selection of *EMEs* during silkworm domestication and breeding phases, identified five strongly selected genes, of which two harbor selected SNPs and Indels within their upstream regulatory region. Assessment of transcription levels indicated a role of them in reproductive changes during the domestication of the silkworm.

## Results

### Genome-wide identification and phylogenetic analysis of *BmEMEs*

**Fig. 1:**
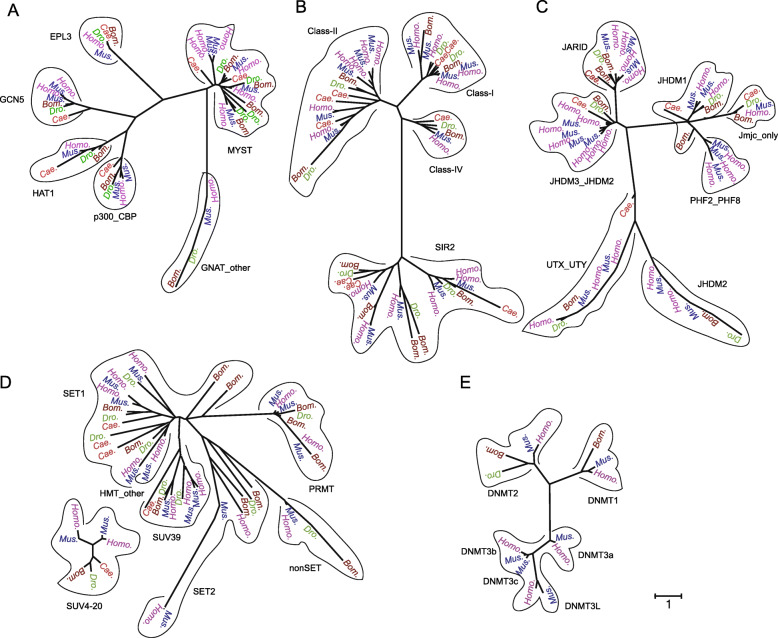
Molecular phylogenetic analysis and classification of *EMEs* in five model animals. **a** HATs. **b** HDACs. **c** HDMs. **d** HMTs. **e** DNMTs, from five species including *Cae.*: *Caenorhabditis elegans, Dro.*: *Drosophila melanogaster*, *Bom.*: *Bombyx mori*, *Mus.*: *Mus musculus*, and *Homo.*: *Homo sapiens*. The trees were constructed using MEGA7 as described in Methods and based on *EMEs* amino acid sequences. The subfamilies are indicated by loops

We identified 41 *BmHMEs*, including 9 *histone acetyltransferase* (*HATs*), 11 *histone deacetylase* (*HDACs*), 13 *histone methyltransferase* (*HMTs*), and 8 *histone demethylase* (*HDMs*) in the silkworm genome by homology searching. Furthermore, two known *DNA methyltransferase*s (*DNMTs*) [7] and one methyl-binding domain protein gene have been previously reported (Additional file 2: Table S2). Thus, 44 *BmEMEs* were included in the present study. Phylogenetic analysis of *EMEs* in five model animals showed that *HATs*, *HDACs*, *HMTs*, *HDMs,* and *DNMTs* could be classified into 6, 4, 7, 7, and 3 subfamilies, and genes in a subfamily among different animals are clustered together, indicating that *EMEs* have high sequence conservation among several species (Fig. 1). Except for the SUV4–20 subfamily of *HMTs*, other subfamilies in the *HAT*, *HDAC*, *HMT*, *HDM,* and *DNMT* families clustered together in the corresponding phylogenetic tree, suggesting their close relationship and similar function. We also noticed that family expansions occurred in some subfamilies such MYST in *HATs*, Class II and SIR2 in *HDACs*, and SET1 in *HMTs*, especially in mouse and human (Fig. 1).

### Similar tissue expression pattern of *EMEs* in different animals

**Fig. 2:**
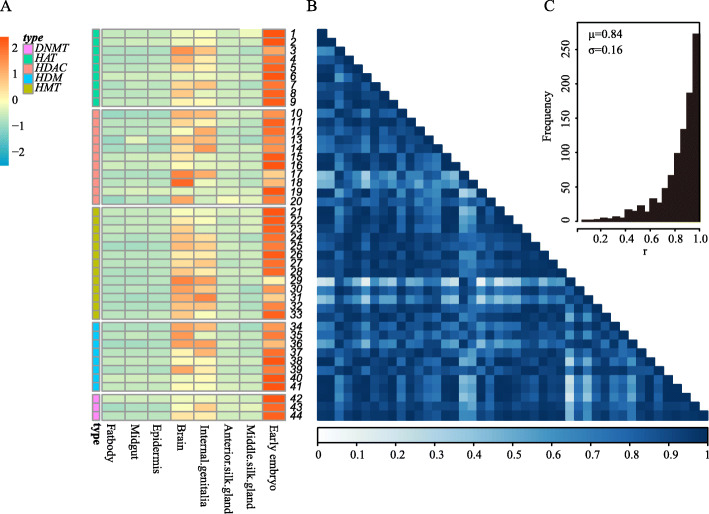
Tissue expression profiles of *BmEMEs*. **a** Heat map of tissue expression patterns of *BmEMEs*. The numbers on the right are the Gene Serial Numbers, which are also shown in Additional file 2: Table S2. **b** Correlation heat map of the expression level of *BmEMEs*. x-axis: Gene Numbers 1–44, from left to right; y-axis: Gene Numbers 1–44, from top to bottom. Each cell represents the Pearson correlation coefficient (r) of the expression levels between gene pairs of corresponding *BmEMEs*. The darker the color, the higher the correlation. **c** Frequency histogram of the Pearson correlation coefficient (r) of *BmEME* expression levels. μ and σ are the mean and standard deviation of Pearson correlation coefficient (r)

To elucidate the role of EMEs in regulating the development of *B. mori*, we investigated the tissue expression patterns of *BmEMEs* using the RNA-seq data from SilkBase v2.1 [23]. The results show that most *BmEMEs* display a high similarity in expression profile (Fig. 2a, Additional file 3: Table S3A). We calculated the correlation coefficient of the expression pattern among *BmEMEs*, and the results showed that the average Pearson correlation coefficient (r) is 0.84, and more than 73% of the gene pairs have an expression pattern similarity (r) > 0.8 (Fig. 2b and c). Furthermore, these are mainly expressed in silkworm embryos, as well as the head and internal genitalia of larvae (Fig. 2a). These findings suggest that *BmEMEs* may be vital to early embryonic development and nervous system and internal genital maturity in silkworm.

To investigate whether this expression feature of *BmEMEs* is universal, we further analyzed the tissue expression patterns of *EMEs* in two model animals, *Drosophila melanogaster* and *Mus musculus*. Similarly, *DmEMEs* and *MuEMEs* also exhibited similar expression patterns. *DmEMEs* are highly expressed in embryos, testes, and ovaries (Additional file 4: Figure S1A, Additional file 3: Table S3B). The *MuEMEs* are also highly expressed in the brain and testis (Additional file 4: Figure S1B, Additional file 3: Table S3C). These results confirm that the expression of *EMEs* is universal among model organisms and indicate that EMEs establish epigenetic modifications and may play an important and conserved role in the development of these tissues, particularly the reproductive system.

### *BmEMEs* undergo selection during domestication and breeding

**Fig. 3:**
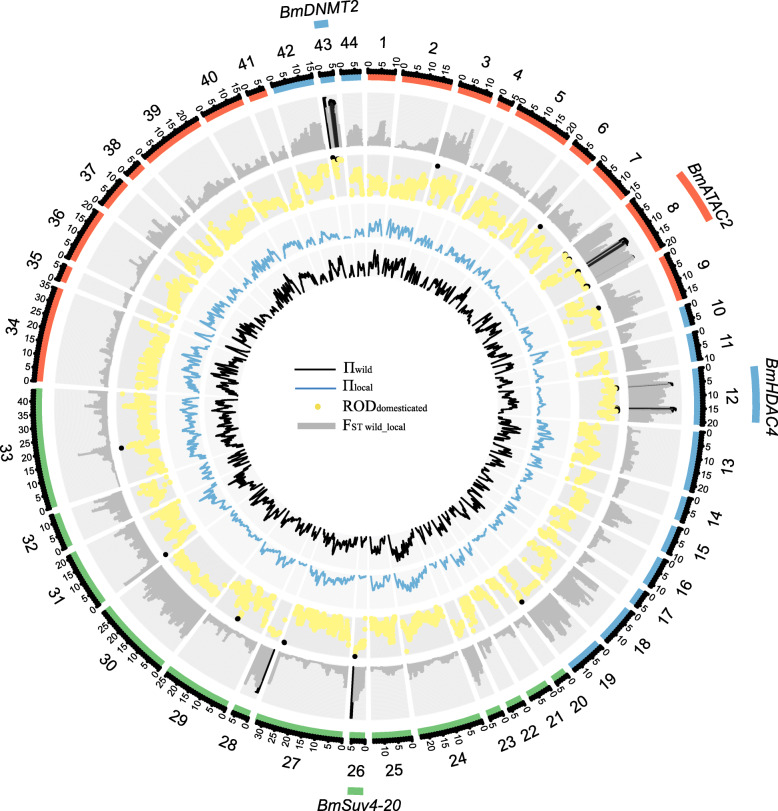
Selective sweeping of *BmEMEs* during domesticated phases of the silkworm. Forty-four *BmEMEs*, 1–44, are grouped and represented by different colors, based on Additional file 2: Table S2. The scale number *1000 is equal to the length (bp) of the gene region. From the outer ring to the inner ring, are histograms of F_ST_ between wild and local silkworm and plots of ROD_domesticated_ and lines of π_local_ and π_wild_. The range of the y-axis of the histogram, plot, and lines is 0–0.9, 0–1, 0–0.05, and 0–0.05, respectively. Histograms of F_ST_ and plots of ROD in black together indicate the identified selective sweeps associated with domestication, i.e., the windows of the highest 1% F_ST wild_local_, the highest 1% of ROD_domesticated_, and the lowest 5% of π_local_ (π_local_ < 0.00095, F_ST wild_local_ > 0.75095, and ROD_domesticated_ > 0.939). The gene with names indicates potential selected genes, which include the selective sweeps

To determine whether *BmEMEs* were under selection during domestication and breeding, we conducted selective sweeping by estimating the sequence divergence (F_ST_), nucleotide diversity (π), and reduction of nucleotide diversity (ROD) in 1-kb windows, targeting the genomic region bearing the 44 *BmEMEs*. F_ST_ and π are the two most commonly used signatures in measuring selective signatures. We first evaluated these indexes as a whole during silkworm domestication and breeding. The maximum and average F_ST_ between wild and local silkworm is 0.881 and 0.185, while that between local and improved silkworm is 0.263 and 0.049, respectively. The average π of *BmEMEs* in wild, local, and improved silkworm is 0.016, 0.009, and 0.008, and the average ROD_domesticatied_ and ROD_breeding_ are 0.39 and 1, respectively. These indicated that the divergence of *BmEMEs* between wild and local silkworm is higher than that between local and improved strains. By taking the top 1% of the highest F_ST_ and ROD values, and the top 5% of low π_local_ as the cutoff, we identified the selective signatures of *BmEMEs* during domestication. We detected strong selection signatures in four *BmEMEs* regions of the domestic silkworm, including *BmATAC2*, *BmHDAC4*, *BmSuv4–20*, and *BmDNMT2* (Fig. 3). With the same threshold, *Bmsirt6* was determined to have undergone selection in improved silkworm (Additional file 5: Figure S2).

### Variants undergo the selection of selected *BmEMEs*

**Fig. 4:**
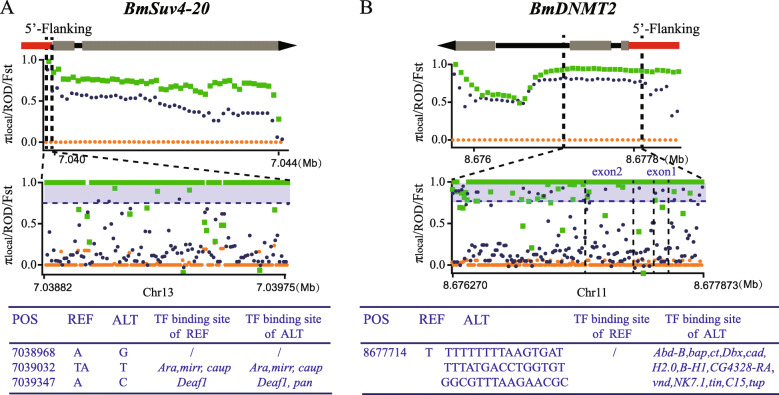
Single variant site selective sweeping analysis. **a** Detailed artificial selection analysis and predicted TF binding on selected variants of *BmSuv4–20*. **b** Detailed artificial selection analysis and predicted TF binding on selected variants of *BmDNMT2*. **Top:** Diagram for gene structure and artificial selection analysis of *BmSuv4–20* (**a**) and *BmDNMT2* (**b**) in 1-kb windows. Orange, deep blue, and green plots in each window represent the average value of π_local_, F_ST wild_local_, and ROD_domesticated_, respectively. The x-axis represents the physical location of the genomic region of *BmSuv4–20* (**a**) and *BmDNMT2* (**b**). The selected regions are situated between the two dotted lines. **Middle:** Details of selection signatures of each variant in selected regions. 3 and 1 variants were detected with significant selection in the 5′-flanking region of *BmSuv4–20* (A) and *BmDNMT2* (B), respectively. Gray shaded areas indicate sites with strong selection signatures (π_local_ < 0.00095, F_ST wild_local_ > 0.75095, and ROD_domesticated_ > 0.939). **Bottom:** The difference of predicted TF binding on selected variants between reference and altered genotypes. POS: genomic position of the variant; REF: reference genotype of the variant. ALT: altered genotype of the variant. “/” indicates no predicted TF-binding sites

To further detect the variants correlated to *BmEME* selection during domestication and breeding, we calculated F_ST_, ROD, and π of each single nucleotide polymorphism (SNP) and insertion and deletion (Indel) in the selected region of *BmEMEs* (Additional file 6: Table S4). We detected a synonymous mutation in exon 9 of *BmHDAC4*, a synonymous mutation and a non-synonymous mutation in exon 1 of *Bmsirt6*, all of which do not cause any change in the functional domain (Additional file 6: Table S4). In addition, 3 and 1 variants were detected with significant selection in the 5′-flanking region of *BmSuv4–20* and *BmDNMT2*, respectively (Fig. 4a and b, Additional file 6: Table S4). By predicting the transcription factor (TF)-binding sites of these variants, we detected the SNP in upstream of *BmSuv4–20*, at Bomo_Chr13: 7,039,347 bp, with genotypes C and A in the wild silkworm population, and the ratio of allele frequency of C:A = 0.65:0.35. However, genotype A was fully fixed in the local silkworm population, leading to a deletion of the TF-binding site *pan* in the local silkworm (Fig. 4a). We also found the Indel in upstream of *BmDNMT2*, at Bomo_Chr11: 8,677,714 bp which comprises two alleles in the wild silkworm strains, the 44-bp insertion type and the reference type, with the ratio of 0.5:0.5. The reference type was fully fixed in local silkworm strains, and the decrease in the allele frequency of the insertion type in local silkworm causes the loss of 13 TF-binding sites (Fig. 4b). These suggested that *BmEMEs* are more likely to be selected at the regulatory sequence, rather than the coding region, and are responsible for the observed difference in TF-binding sites between wild and local silkworm.

### The expression pattern of *BmSuv4–20* and *BmDNMT2* is correlated with silkworm internal genitalia development

**Fig. 5:**
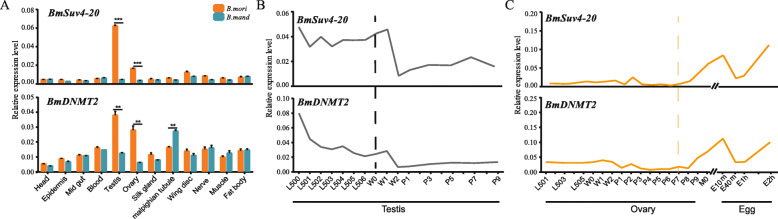
Spatiotemporal expression of *BmSuv4–20* and *BmDNMT2*. **a** Relative expression of *BmSuv4–20* and *BmDNMT2* in the 3rd-d of fifth instar stage of larvae of *Bombyx mandaina* and *B. mori*. **b**, **c** Temporal expression profiling of these genes during testis (**b**) and ovary (**c**) development. *BmSuv4–20* and *BmDNMT2* are highly expressed during the meiotic division of spermatocytes, from the start of the fifth instar stage of the larvae to the start of wandering (L500–W0) (**b**) and the meiotic division of oocytes, from the 7th d of pupa to 2 h after laying eggs (P7–E2h) (**c**). L500–L506: The start to the 6th d of the fifth instar stage of larvae, W0–W2: The start to the 2rd d of the wandering stage of larvae, P1–P9: The 1st d to the 9th d of the pupa, M0: The start of moth, and E10m–E2h: 10 min to 2 h after laying eggs. *Sw22934* was used as a reference; three replicates were used per time point

To verify whether the mutation upstream of *BmSuv4–20* and *BmDNMT2* causes differential expression between wild and domestic silkworm, we assessed tissue expression in the 3rd-d of fifth instar stage of *B. mandaina* and *B. mori* larvae. *BmSuv4–20* was specifically expressed in internal genitalia, whereas *BmDNMT2* showed a relatively broad-spectrum expression pattern in various tissues. Notably, the expression of both genes was significantly higher in the testes and ovaries of *B. mori* than *B. mandaina* (*P* < 0.001, *P* < 0.01) (Fig. 5a). Thus, we assessed their expression profiles during testes and ovary development in *B. mori*. In the testes, primary spermatocytes generally enter meiosis at the end of the third instar stage or the beginning of the fourth instar larval stage of silkworm, and after two rounds of meiosis, sperm cells are formed before the larvae enter into wandering stage mostly. Then, sperm cells transform into mature sperm during the pupa stage. Our temporal expression profiling showed that *BmSuv4–20* and *BmDNMT2* are highly expressed during the meiotic division of sperm, from the start of the fifth instar stage of the larvae to the start of wandering, but was reduced subsequently in the pupa stage (Fig. 5b). While the eggs enter meiosis 1–2 d before mothing, after mating, the first phase of meiosis was completed around 40 min after laying eggs. Then, the second phase of meiosis started 60 min after laying eggs and is completed within 2 h after laying eggs, forming a complete egg. Temporal expression profiling showed that these two genes are highly expressed during the meiotic division of eggs (Fig. 5c). These results implied that *BmSuv4–20* and *BmDNMT2* are correlated with the meiosis of sperm and egg cells of domestic silkworm and participate in incurring changes in reproductive characteristics during silkworm domestication.

## Discussion

The transformation to an indoor breeding environment and artificial selection have created a huge difference in a series of physiological traits between wild and domesticated silkworm. For example, compared to wild silkworm, domesticated silkworm has higher fertility but slower growth rates [24, 25]. For stress resistance, wild silkworm shows strong resistance to adverse environments (pesticides, ultraviolet radiation) [26], but its resistance to pathogens (viruses, bacteria and fungi) is generally thought to be lower than domesticated silkworms [27]. Interestingly, this study has determined that two *BmEMEs*, namely, *BmSuv4–20* and *BmDNMT2*, harbor strong selection signals during artificial selection in domestication. Meanwhile, these genes are highly expressed in the testis and ovary of domesticated silkworm compared to the wild silkworm and may be involved in the regulation of germ cell meiosis. We deduce that these contribute to changes in reproductive characteristics during silkworm domestication.

Suv(ar)4–20 is an enzyme that catalyzes histone H4K20 demethylation (H4K20me2) and trimethylation (H4K20me3) from H4K20 monomethylation (H4K20me1) [28]. These three states of histone H4 lysine 20 methylation (H4K20me) are important to basic biological processes such as DNA replication [29], DNA damage repair [30], and pericentric heterochromatin formation [31]. *Suv4–20 h* mutant mice exhibit perinatal lethality [32]. Despite the lack of evidence showing that this gene is functionally related to reproduction, H4K20me1 plays a role in mouse oocyte meiotic maturation [33]. Our results show that the expression profile of Suv4–20 coincides with gamete formation in silkworm. This implies that Suv4–20 and H4K20me2/me3 are involved in reproductive system development and contribute to changes in reproduction ability during silkworm domestication. However, further functional validation of these findings is warranted.

*Dnmt2* is another *EME* that was strongly selected during silkworm domestication. Although it is the most conserved member of DNA methyltransferases [34], the function of *Dnmt2* remains unclear. Its major function involves highly specific tRNA methyltransferase activity of tRNA^Asp^ [35] and several other tRNAs [36], thus playing a role in protecting tRNA against fragmentation and protein translation [37, 38]. However, Dnmt2 is the sole cytosine DNA methyltransferase in *Drosophila* that influences genomic methylation. Research studies involving *Drosophila* have revealed its function in retrotransposon silencing, telomere integrity, stabilization of repeats, and longevity and stress response [39, 40]. *Dnmt2* is also activated during bacterial infection and affects its proliferation in *Helicoverpa armigera* [41]. Recently, Zhang reported that functional deletion of Dnmt2 in mouse abolishes intergenerational transmission of sperm small non-coding RNAs (sncRNA) [42]. In *Nilaparvata lugens*, RNA interference-mediated depletion of *NlILP2* or *NlILP4* dramatically decreased the expression levels of *NlDnmt2*, resulting in severely impaired ovary growth as well as substantial reduction in fecundity [16], indicating that *Dnmt2* plays a role in ovary development. We show that *Dnmt2* is differentially expressed between the testes and ovaries of domestic and wild silkworm. This observation, combined with its function, suggests that *Dnmt2* plays a role in inner reproductive system development in silkworm and undergoes selection during domestication.

Our results show that *BmSuv4–20* and *BmDNMT2* have incurred selective mutations in their upstream regions (Fig. 4). Expression profiling indicates that these may be vital for the development of early embryos, as well as the nervous and reproductive systems. Mutations causing functional loss of *BmEMEs* may lead to severe adaptation deficiency, which eventually is eliminated during artificial selection. Thus, rational speculation is that the variants in the upstream region of *BmEMEs* are more likely to be selected because the effect of these variants may be mild. They may only cause differential gene expression between wild and domesticated silkworm (Fig. 5), which then further alters the epigenetics modification landscape of silkworm germ cells during domestication, ultimately resulting in diverse reproductive characters. Genomic changes influence the expression of *BmEMEs* in all the tissues; however, our results show that *BmSuv4–20* and *BmDNMT2* are only differentially expressed in certain tissues such as the testis and ovary. We have shown that selective mutations in their upstream regions result in the loss of predicted TFs in *B. mori* (Fig. 4). We first assessed whether these TFs are responsible for the observed tissue-specific differential expression of these two genes. The expression of these TFs in the head, testis, ovaries, and nerves of wild silkworm and domesticated silkworm was investigated and revealed that only *BmDbx* is highly expressed in these tissues, and the highest levels were observed in the ovary and nerves of wild silkworm (Additional file 6: Figure S3). *Dbx* is a homeodomain-containing transcription factor. In flies, it contributes to the development of specific subsets of interneurons by cross-repressive, lineage-specific interactions with eve and hb9 (exex), the motoneuron-promoting factors [43]. Expression profiling shows that in the domestic silkworm, *Dbx* has a lower expression leave in the ovary and nerves, and its binding site was also lost. This indicates that *Dbx* may also have the ability to repress the expression of *DNMT2*, and with binding site loss, *DNMT2* may then be upregulated in the ovary of domestic silkworm (Fig. 5a). However, this still cannot explain why *DNMT2* is upregulated in the testis of domestic silkworm. In addition, the reason why *BmSuvv4–20* is specifically upregulated in the testis and ovary of *B. mori* remains unknown. Another possibility is that these variations may change the epigenetic markers or with other regulators in the upstream region of the two genes. However, these findings must be validated by further experiments.

## Conclusions

Here, we comprehensively investigated *EMEs* in the whole genome of silkworm and identified two *BmEMEs*, namely, *BmSuv4–20* and *BmDNMT2*, which are strongly selected during silkworm domestication and expressed higher in the testes and ovaries of the domesticated silkworm than wild silkworm. Their expression pattern is correlated with sperm and egg cell meiosis. Thus, we report that *BmSuv4–20* and *BmDNMT2* may be involved in incurring changes in reproductive characters during domestication. Our findings may serve as a reference for further understanding the role of epigenetic modifications during animal domestication.

## Methods

### Genome-wide identification and tissue expression of *EMEs* in different model animals

To identify *BmHMEs,* the amino acid sequences of *EMEs of Caenorhabditis elegans*, *Drosophila melanogaster*, *Mus musculus,* and *Homo sapiens* from WERAM 1.0 (http://weram.biocuckoo.org/) [44] (Additional file 8) were subjected to analysis using blastp (expect threshold = 10) in SilkBase v2.1 (http://silkbase.ab.a.u-tokyo.ac.jp/cgi-bin/index.cgi) [23]. All *EMEs* homologs in silkworm and four other model animals are shown in Additional file 2: Table S2. Then, phylogenetic reconstruction was performed using MEGA7 (https://www.megasoftware.net/) [45], and the *EME* sequences of these five species were aligned using ClustalW. The trees were generated using the neighbor-joining method based on the Jones-Taylor-Thornton (JTT) matrix-based model, and bootstrapping was used for phylogeny testing with 2000 replications. The bootstrap values were added in all presented phylogenetic trees. The generated trees were confirmed by Bayesian analyses using MrBayes 3.2.7a (http://nbisweden.github.io/MrBayes/) [46].

To investigate the tissue expression of *EMEs* in different model animals, we downloaded RNA-Seq RPKM/TPM values of tissues of *B. mori*, *D. melanogaster*, and *M. musculus* from SilkBase v2.1 (Additional file 3: Table S3A), FlyBase 2.0 (http://flybase.org/) [47] (Additional file 3: Table S3B) and Expression Atlas (https://www.ebi.ac.uk/gxa/home) [48] (Additional file 3: Table S3C). Then, we calculated the correlation coefficient between the expression values of different *BmEMEs* for visualization using function cor.test () of R version 3.5.2 (https://www.r-project.org).

### Selective sweeping of *BmEMEs* during domestication and breeding

To explore the selective sweep regions of *BmEMEs* in local and improved silkworm, we divided the 136 resequenced silkworm lines [49] into three groups as wild, local, and improved populations, and deleting a few polyvoltine improved Chinese strains (Additional file 9: Table S5). After filtering the sites with a miss rate of > 20% in all strains, we detected a total of 47,766 variants in the genomic regions that were related to gene body and upstream 2-kb of 44 *BmEMEs* [49] (Additional file 10). Using a sliding-window method (1-kb windows with 100-bp sliding steps), the gene-related sequence divergence (F_ST_) between wild and local populations, as well as local and improved populations, and nucleotide diversity (π) of the three groups were calculated. Based on the π value, the reduction of diversity ROD in domesticated lines (ROD_domesticatied_ = 1- π_local_/π_wild_) and in improved lines (ROD_breeding_ = 1-π_improved_/π_local_) in each bin was also calculated. The windows simultaneously containing the top 1% of the high F_ST_ values, the top 1% of the high ROD values, and the top 5% of low π_local_ (domesticated stage) and π_improved_ values (breeding stage) were considered as regions under selection. Genes with the selected regions were regarded as selected genes.

### Screening and annotation of single selected sites within selected *BmEMEs* in domestic and breeding phases

We further calculated the selective pressure signatures: F_ST_, π, and ROD of each mutant in *BmEME* selected regions. Taking the same screening criteria for selected windows, the outliers of these genes were identified as the selected variants. Among these, we searched for the SNPs and Indels located in the gene coding region and 5′ flanking region. For non-synonymous mutations in gene coding region, we predicted their protein domain change in Pfam (http://pfam.xfam.org/) [50]; for mutations in gene 5′ flanking regions, we used the 60-bp genomic sequence, including the mutation site, and predicted changes in TF-binding profiles by scanning with all insecta matrix models in JASPAR 2020 (http://jaspar.genereg.net/) [51].

### Spatiotemporal expression of target genes

We used real-time PCR to quantify expression levels. The most widely used strain, 19–200, preserved at the State Key Laboratory of Silkworm Biology of Southwest University, was used as domestic strain. Wild silkworms were sampled in 2017 in Beibei, Chongqing, China. Silkworm tissues were collected from 3rd d of the fifth instar stage of larvae and used for tissue expression analysis of target genes and related transcription factors in *B. mandaina* and *B. mori*. The testis from the start of the fifth instar stage of larvae to the 9th d of pupa, ovary from 1st-d of fifth instar stage of larvae to the start of the moth and egg stages at 10 min, 40 min, 1 h, and 2 h after spawning of 19–200 were collected for assessment of temporal expression of genes in these tissues. Total RNA was extracted with the RNApure Kit (BioTeke, Beijing, China) and digested with DNase I (Takara, Japan) to remove any remaining DNA. Complementary DNA was synthesized using the PrimeScript RT reagent kit (Takara, Japan). The sequences of the primers used in real-time PCR analysis are presented in Additional file 8: Table S5. *Eukaryotic translation initiation factor 4A* (*Eif-4a*; XR_001139998) was used as a reference gene. Real-time PCR was performed in triplicate with SYBR Green PCR Mix (Bio-Rad, Hercules, CA) reagents and subjected to the CFX96TM Real-Time PCR Detection System (Bio-Rad, Hercules, CA). The reaction system was 10 μL, and there were three technical replicates per sample. Messenger RNA abundance of each gene was calculated using the 2^-△△CT^ method and normalized to *Eif-4a*.

## Supplementary information

**Additional file 1 **: **Table S1.** Positively selected *EMEs* in domesticated animals.

**Additional file 2 **: **Table S2.** Genome-wide identification of *BmEMEs* and their orthologues in *Caenorhabditis elegans*, *Drosophila melanogaster*, *Mus musculus* and *Homo sapiens.*

**Additional file 3 **: **Table S3.** The RNA-seq data of *EMEs* in silkworm, *Bombyx mori* (A), *Drosophila melanogaster* (B) and *Mus musculus* (C).

**Additional file 4 **: **Figure S1.** The tissue expression profile of *EMEs* of *Drosophila melanogaster* (A) and *Mus musculus* (B). The Gene Numbers on the right are from WERAM 1.0 database and Ensembl^ASIA^.

**Additional file 5 **: **Figure S2.** Selective sweeping of *BmEMEs* during breeding phases of silkworm. 44 *BmEMEs*, 1–44, are grouped and represented by different colors, related to Additional file 2: Table S2. The scale number *1000 equal to the length (bp) of the gene region. From the outer ring to the inner ring, are the F_ST_ histograms between local and improved silkworm, plots of ROD_breeding_ and lines of π_improved_ and π_local_. Range of y-axis of the histogram, plot, and lines are 0–0.3, 0–1, 0–0.05, and 0–0.05. Histograms of F_ST_ and plots of ROD in black together indicate the identified selective sweeps associated with breeding, i.e., the windows of the highest 1% F_ST local_improved_, the highest 1% of ROD_breeding_ and the lowest 5% of π_improved_ (π_improved_ < 0.000256, F_ST local_improved_ > 0.2002 and ROD_breeding_ > 0.9872). The gene with names indicates potential selected genes, which include the selective sweeps.

**Additional file 6 **: **Table S4.** Single mutant sites selected in *BmEMEs* under selection.

**Additional file 7 **: **Figure S3. (A)** Relative expression of transcription factors in the head, testis, ovaries, and nerves of 3rd-d of the fifth instar stage of larvae of *B. mandaina* and *B. mori*, which was predicted in the upstream of *BmSuv4–20* and *BmDNMT2*. **(B)** Tissue expression pattern of transcription factors *Bmct* and *BmDbx* in the 3rd-d of the fifth instar stage of larvae of *B. mandaina* and *B. mori***.** *Sw22934* was used as a reference; three replicates were used per time point.

**Additional file 8 **The amino acid sequences of *EMEs of Caenorhabditis elegans*, *Drosophila melanogaster*, *Mus musculus*, and *Homo sapiens* for homological blasting.

**Additional file 9 **: **Table S5.** The strains of wild, local, and improved populations, respectively.

**Additional file 10 **SNPs data of the genomic regions of *BmEMEs* in wild, local, and improved silkworm populations.

**Additional file 11 **: **Table S6.** Primers used in real-time PCR analysis.

## Data Availability

The datasets supporting the conclusions of this study are included within the article and as additional files.

## Abbreviations

EMEs: Epigenetic modifying enzymes
PTMs: Post-translational modifications
*HMEs*: *Histone modification enzymes*
*DNMTs*: *DNA methyltransferases*
*HATs*: *Histone acetyltransferase*
*HDACs*: *Histone deacetylase*
*HMTs*: *Histone methyltransferase*
*HDMs*: *Histone demethylase*
*Cae.*: *Caenorhabditis elegans*
*Dro.*: *Drosophila melanogaster*
*Bom.*: *Bombyx mori*
*Mus.*: *Mus musculus*
*Homo.*: *Homo sapiens*
SNP: Single nucleotide polymorphism
Indel: Insertion and deletion
TF: Transcription factor
JTT: Jones-Taylor-Thornton
*Eif-4a*: *Eukaryotic translation initiation factor 4A*
