# Supplementary figures and images for: Identification, expression, and artificial selection of silkworm epigenetic modification enzymes
Source: BMC Genomics. 2020 Oct 23;21:740. doi: 10.1186/s12864-020-07155-z (PMC7585183; doi:10.1186/s12864-020-07155-z)

A

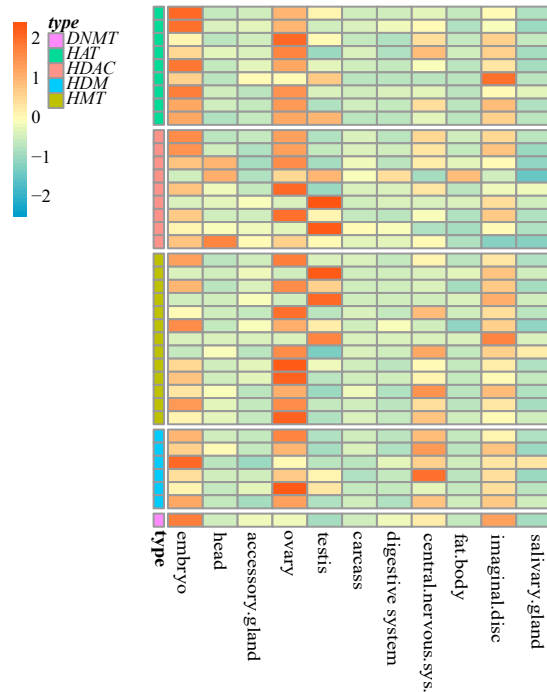

B

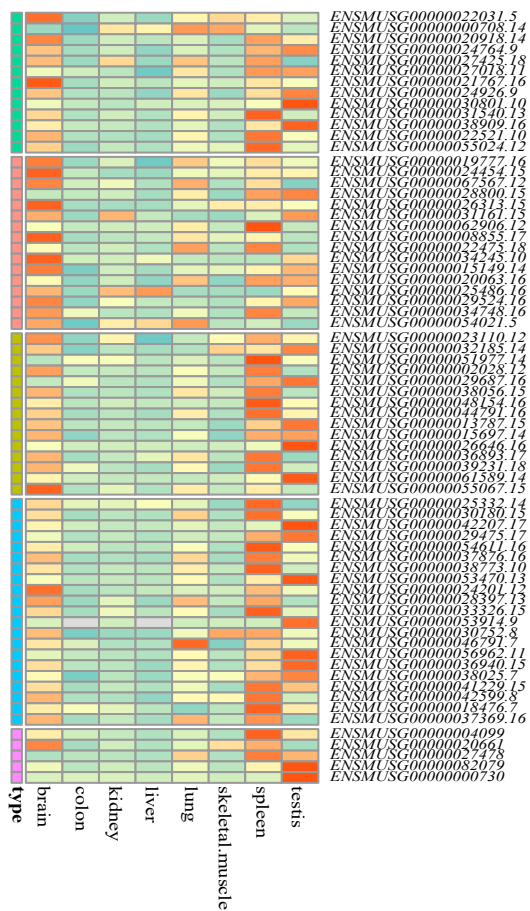

Supplement: Supplementary file 4 — Additional file 4 : Figure S1. The tissue expression profile of EMEs of Drosophila melanogaster (A) and Mus musculus (B). The Gene Numbers on the right are from WERAM 1.0 database and EnsemblASIA. [file 12864_2020_7155_MOESM4_ESM.pdf]

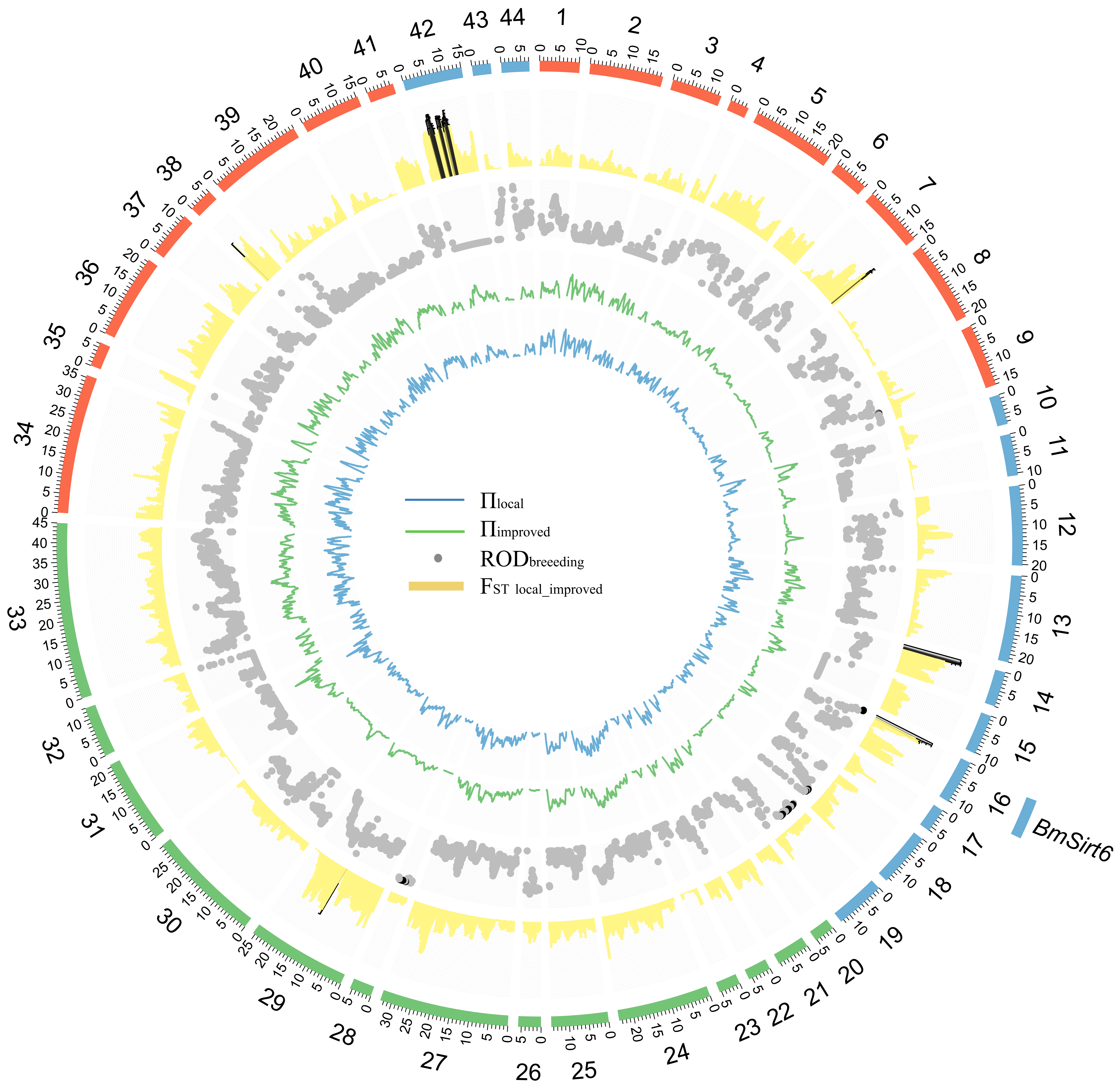

Supplement: Supplementary file 5 — Additional file 5 : Figure S2. Selective sweeping of BmEMEs during breeding phases of silkworm. 44 BmEMEs, 1–44, are grouped and represented by different colors, related to Additional file 2: Table S2. The scale number *1000 equal to the length (bp) of the gene region. From the outer ring to the inner ring, are the FST histograms between local and improved silkworm, plots of RODbreeding and lines of πimproved and πlocal. Range of y-axis of the histogram, plot, and lines are 0–0.3, 0–1, 0–0.05, and 0–0.05. Histograms of FST and plots of ROD in black together indicate the identified selective sweeps associated with breeding, i.e., the windows of the highest 1% FST local_improved, the highest 1% of RODbreeding and the lowest 5% of πimproved (πimproved < 0.000256, FST local_improved > 0.2002 and RODbreeding > 0.9872). The gene with names indicates potential selected genes, which include the selective sweeps. [file 12864_2020_7155_MOESM5_ESM.pdf]

A

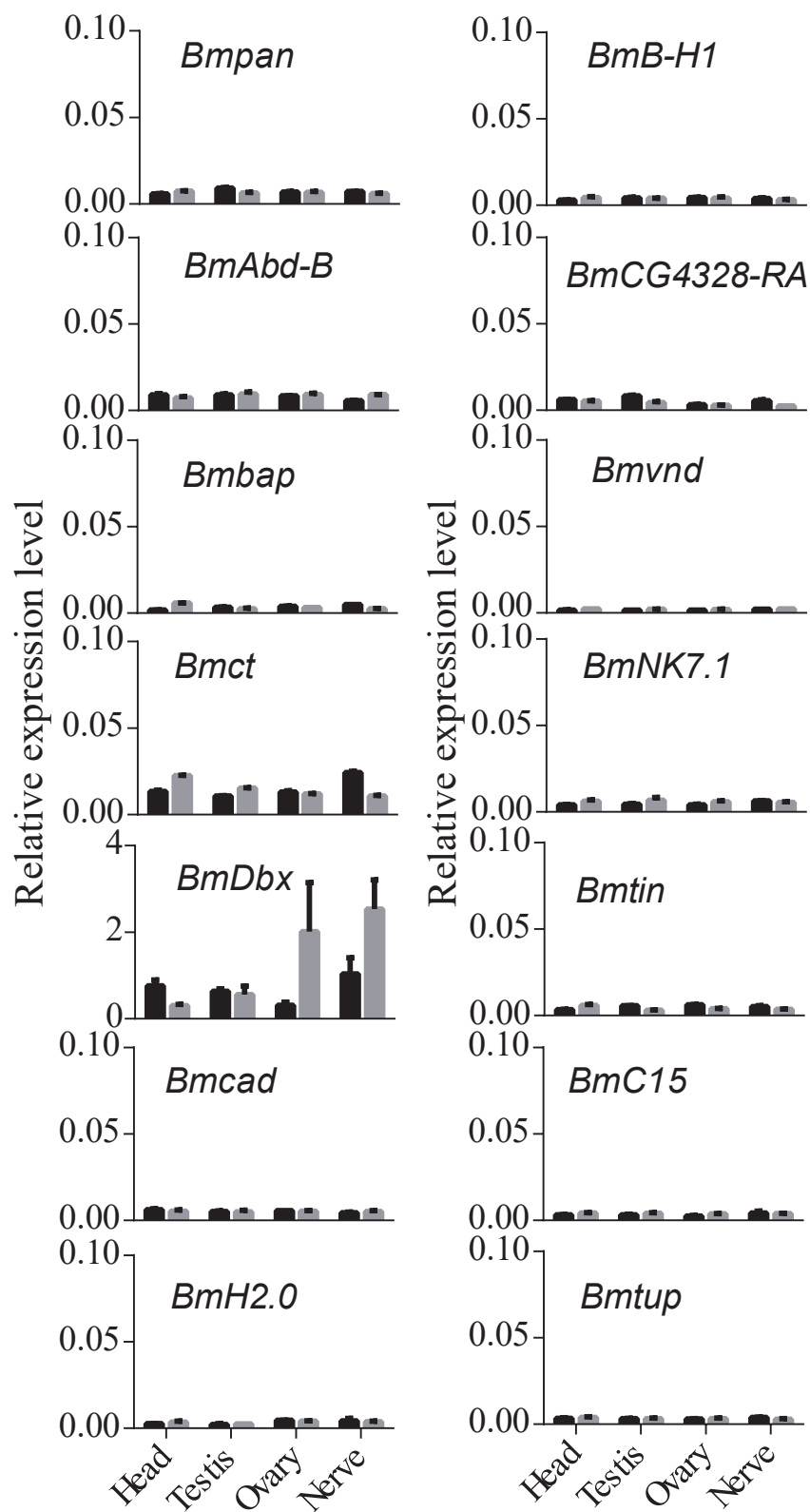

B

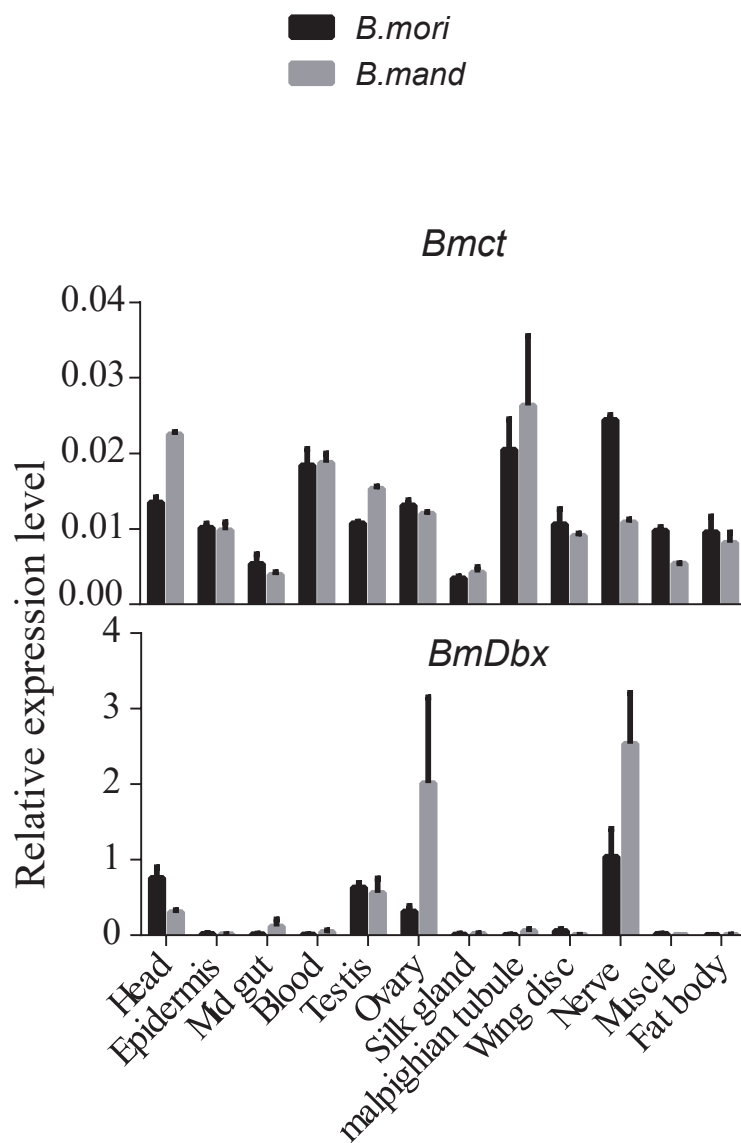

Supplement: Supplementary file 7 — Additional file 7 : Figure S3. (A) Relative expression of transcription factors in the head, testis, ovaries, and nerves of 3rd-d of the fifth instar stage of larvae of B. mandaina and B. mori, which was predicted in the upstream of BmSuv4–20 and BmDNMT2. (B) Tissue expression pattern of transcription factors Bmct and BmDbx in the 3rd-d of the fifth instar stage of larvae of B. mandaina and B. mori. Sw22934 was used as a reference; three replicates were used per time point. [file 12864_2020_7155_MOESM7_ESM.pdf]
